# Supplementary material for: Analysis of p67 allelic sequences reveals a subtype of allele type 1 unique to buffalo-derived Theileria parva parasites from southern Africa
Source: PLoS One. 2020 Jun 29;15(6):e0231434. doi: 10.1371/journal.pone.0231434 (PMC7323972; doi:10.1371/journal.pone.0231434)
Supplement: S1 Table — (DOCX) [file pone.0231434.s002.docx]

**S1 Table.** The Ct values of samples from active clinical cases of Corridor disease and non-clinical *T. parva*-positive cases collected from Mpumalanga province in South Africa.

| **^a^Clinical cases of Corridor disease** | | | **^b^Non-clinical *T. parva*-positive cases** | | | |
| --- | --- | --- | --- | --- | --- | --- |
| Sample ID | Origin | qPCR (Ct) | Sample ID | Origin | **^c^**qPCR (Ct) | **^d^**qPCR (Ct) |
| C3 | Utha A | 26.84 | F25 | Welverdiend A | 34.13 | negative |
| C10 | Clare A | 25.71 | F79 | Welverdiend B | 34.57 | positive (36.4) |
| C66 | Hlalakahle | 25.74 | F238 | Hlalakahle | >40 | negative |
| C71 | Hlalakahle | 23.86 | F246 | Hlalakahle | 36.81 | negative |
| C80 | Welverdiend B | 24.85 | F268 | Hlalakahle | 36.77 | negative |
| C81 | Thlavekisa | 22.92 | F287 | Hlalakahle | 37.04 | negative |
| C84 | Islington | 29.17 | F312 | Hlalakahle | 36.63 | NS |
| C89 | Hlalakahle | 25.42 | F328 | Seville B | 36.03 | negative |
| C91 | Utha A | 22.88 | F369 | Islington | 27.01 | NS |
| C108 | Hlalakahle | 21.75 | F376 | Islington | 33.63 | negative |
| C109 | Hlalakahle | 24.64 | F300 | Hlalakahle | 31.69 | NS |
| C110 | Seville B | 26.41 |  |  |  |  |
| C129 | Hlalakahle | 23.87 |  |  |  |  |
| C133 | Hlalakahle | 29.02 |  |  |  |  |

Ct - refers to the ‘cycle threshold’, which is the cycle at which fluorescence from amplification exceeds the background. A lower Ct value correlates with a higher starting concentration of target DNA in a sample and vice versa.

NS - Not sampled.

F300 was not used in the current study due to inadequate DNA.

**^a^** Samples collected from active clinical cases of Corridor disease in Hluvukani, Mnisi community, Mpumalanga province. They had low Ct values (<30) indicative of high infection levels.

**^b^** Samples forming part of 432 collected from apparently healthy cattle from a herd with previous *T. parva* infections within the Mnisi Community, Mpumalanga province.

**^c^** *Theileria parva* DNA was detected in 11 samples (2.6%) (shown in the table) out of the 432 with high Ct values (>30) characteristic of very low infection levels. *Theileria parva* detection was performed using the *T. parva*-specific qPCR assay (Sibeko *et al*., 2008).

**^d^** Eight out of the eleven non-clinical *T. parva*-positive cases were resampled after six months and only one case was still positive.
