# Supplementary material for: Analysis of p67 allelic sequences reveals a subtype of allele type 1 unique to buffalo-derived Theileria parva parasites from southern Africa
Source: PLoS One. 2020 Jun 29;15(6):e0231434. doi: 10.1371/journal.pone.0231434 (PMC7323972; doi:10.1371/journal.pone.0231434)
Supplement: S2 Table — (DOCX) [file pone.0231434.s003.docx]

**S2 Table.** Predicted protein sequence alignment of allele type 2 identified in *T. parva* parasites from cattle and buffalo

| Host | ^a^Sequence ID/Accession number | ^b^Predicted protein sequence |
| --- | --- | --- |
| Cattle | **TpM12**  **KY912965** EDSTVSTDVSPTIPTPVSEEIITPTLQAQTKEEVPPADLSDQVPSNGSDSEEEDNKS-TS 198  **KY912967** EDSTVSTDVSPTIPTPVSEEIITPTLQAQTKEEVPPADLSDQVPSNGSDSEEEDNKS-TS 198  KNP_MN_C108_9 EDSSLGTDVPQSISTPVSEEIITPTLQAQTKEEVPPADLSDQVPSNGSDSEEEDEDS-SL 198  KNP_MN_C84_2 EDSSLGTDVPQSISTPVSEEIITPTLQAQTKEEVPPADLSDQVPSNGSDSEEEDEDS-SL 198  KNP_MN_C133 EDSTLSTDVSPTIPTPVSEEIITPTLQAQTKEEVPPADLSDQVSSNGSDSEEEDNKS-TS 198  KNP_MN_C3 EDSTLSTDVSPTIPTPVSEEIITPTLQAQTKEEVPPADLSDQVSSNGSDSEEEDNKS-TS 198 | |
| Buffalo | **U40703** EDSTVSTDVSPTIPTPVSEEIITPTLQAQTKEEVPPADLSDQVPSNGSDSEEEDNKS-TS 198  K_Mar_C6 EDSTLSTDISPTIPTPVSEEIITPTLQTQTKEEVPPADLSDQVPSNGSDSEEEDGDS-SL 198  K_Mar_A7 EDSSLGTDVPQSIPTPVSEEIITPTLQAQTKEEVPPADLSDQVPSNGSDSEEEDGDS-SL 198  TZ_T-B8 EDSTVSTDVSPTIPTPVSEEIITPTLQAQTKEEVPPADLSDQVPSNGSDSEEEDNKS-TS 198  TZ_T-B5 EDSTVSTDVSPTIPTPVSEEIITPTLQAQTKEEVPPADLSDQVPSNGSDSEEEDNKS-TS 198  KZN_HIP_D7 EDSSLGTDVPQSIPTPVSEEIITPTLQAQTKEEVPPADLSDQVPSNGSDSEEEDEDS-SL 198  KZN_HIP_B4 EDSSLGTDVPQSISTPVSEEIITPTLQAQTKEEVPPADLSDQVPSNGSDSEEEDEDS-SL 198  Moz_Buf_10 EDSSLGTDVPQSISTPVSEEIITPTLQAQTKEEVPPADLSDQVPSNGSDSEEEDEDS-SL 198  **KY912965** EDSTVSTDVSPTIPTPVSEEIITPTLQAQTKEEVPPADLSDQVPSNGSDSEEEDNKS-TS 198  **KY912967** EDSTVSTDVSPTIPTPVSEEIITPTLQAQTKEEVPPADLSDQVPSNGSDSEEEDNKS-TS 198 | |
| Cattle | **AR22.7**  **KY912965** SKDEKELKKTLQPGKTSTGETTSGQDLNSKQQQTGVSDLASGSHSSGLTVPGVGVPGAVS 258  **KY912967** SKDEKELKKTLQPGKTSTGETTSGQDLNSKQQQTGVSDLASGSHSSGLTVPGVGVPGAVS 258  KNP_MN_C108_9 GTDERNLKKTLQPGKTSTGETTSDQHLKSKQQQTGVSDLASGSHSSGLKVPGVGVPGAVS 258  KNP_MN_C84_2 GTDERNLKKTLQPGKTSTGETTSDQDLKSKQQQTGVSDLASGSHSSGLKVPGVGVPGAVS 258  KNP_MN_C133 SKDEKELKKTLQPGKTSTGETTSGQDLNSKQQQTGVSDLASGSHSSGLKVPGVGVPGAVS 258  KNP_MN_C3 SKDEKELKKTLQPGKTSTGETTSGQDLNSKQQQTGVSDLASGSHSSGLKVPGVGVPGAVS 258 | |
| Buffalo | **U40703** SKDEKELKKTLQPGKTSTGETTSGQDLNSKQQQTGVSDLASGSHSSGLTVPGVGVPGAVS 258  K_Mar_C6 GTDERNLKKTLPPGKTSTGETTSDQDLKSKQQQTGVSDLASGSHSSGLTVPGVGVPGAVS 258  K_Mar_A7 GTDERNLKKTLPPGKTSTGETTSDQDLKSKQQQTGVSDLASGSHSSGLTVPGVGVPGAVS 258  TZ_T-B8 SKDEKELKKTLQPGKTSTGETTSGQDLNSKQQQTGVSDLASGSHSSGLTVPGVGVPGAVS 258  TZ_T-B5 SKDEKELKKTLQPGKTSTGETTSGQDLNSKQQQTGVSDLASGSHSSGLTVPGVGVPGAVS 258  KZN_HIP_D7 GTDERNLKKTLQPGKTSTGETTSDQDLKSKQQQTGVSDLASGSHSSGLKVPGVGVPGAVS 258  KZN_HIP_B4 GTDERNLKKTLQPGKTSTGETTSDQDLKSKQQQTGVSDLASGSHSSGLKVPGVGVPGAVS 258  Moz_Buf_10 GTDERNLKKTLQPGKTSTGETTSDQDLKSKQQQTGVSDLASGSHSSGLKVPGVGVPGAVS 258  **KY912965** SKDEKELKKTLQPGKTSTGETTSGQDLNSKQQQTGVSDLASGSHSSGLTVPGVGVPGAVS 258  **KY912967** SKDEKELKKTLQPGKTSTGETTSGQDLNSKQQQTGVSDLASGSHSSGLTVPGVGVPGAVS 258 | |

**^a^** Reference sequences are bolded. Annotation of other sequence IDs is provided in Figure 1 legend.

**^b^** Amino acid substitutions are highlighted in cyan; TpM12 (TKEEVPPADLSDQVP**)** and AR22.7 (LQPGKTS**)** are B-cell epitopes.
