# Supplementary material for: Analysis of p67 allelic sequences reveals a subtype of allele type 1 unique to buffalo-derived Theileria parva parasites from southern Africa
Source: PLoS One. 2020 Jun 29;15(6):e0231434. doi: 10.1371/journal.pone.0231434 (PMC7323972; doi:10.1371/journal.pone.0231434)
Supplement: S3 Table — (DOCX) [file pone.0231434.s004.docx]

**S3 Table.** Predicted protein sequence alignment of allele type 3 identified in *T. parva* parasites from cattle and buffalo

| Host | ^a^Sequence ID/Accession number | ^b^Predicted protein sequence |
| --- | --- | --- |
| Cattle | **TpM12**  **KY912979** EDSTVSTDVSPTIPTPVSEEIITPTLQAQTKEEVPPASSSD---SEQEDSEENGDNVLKN 198  **KY912995** EDSTLSTDISPTIPTPVSEEIITPTLQTQTKEEVPPASSSD---SEQEDSEENGGDGLKN 198  KNP_MN_C81 EDSTLSTDVSPTIPTPVSEEIITPTLQGQTKEEVPPASGSD---SEQEDSEENEDDVLKN 198  KNP_MN_C108_4 EDSTLSTDVSPTIPTPVSEEIITPTLQGQTKEEVPPASGSD---SEQEDSEENEDDVLKN 198  KNP_MN_C89_1 EDSTLSTDVSPTIPTPVSEEIITSTLQAQTKEEVPPASSSD---SEQEDSEENGNDGLKN 198  KNP_MN_C91 EDSTLSTDVSPTIPTPVSEEIITSTLQAQTKEEVPPASSSD---SEQEDSEENGNDGLKN 198 | |
| Buffalo | **KY912979** EDSTVSTDVSPTIPTPVSEEIITPTLQAQTKEEVPPASSSD---SEQEDSEENGDNVLKN 198  **KY912995** EDSTLSTDISPTIPTPVSEEIITPTLQTQTKEEVPPASSSD---SEQEDSEENGGDGLKN 198  **JX442249** EDSTVSTDVSPTIPTPVSEEIITPTLQAQTKEEVPPASSSD---SEQEDSEENGNDGLKN 198  K_Mar_B6 EDSTVSTDVSPTIPTPVSEEIITPTLQAQTKEEVPPASSSD---SEQEDSEENGDNVLKN 198  K_Mar_D2 EDSTVSTDVSPTIPTPVSEEIITPTLQAQTKEEVPPASSSD---SEQEDSEENGDNVLKN 198  K_Mar_A4 EDSTVSTDVSPTIPTPVSEEIITPTLQAQTKEEVPPASSSD---SEQEDSEENGDNVLKN 198  K_Mar_C2 EDSTVSTDVSPTIPTPVSEEIITPTLQAQTKEEVPPASSSD---SEQEDSEENGDNVLKN 198  TZ_T-A10 EDSTLSTDVSPTIPTPVSEEIITPTLQSQTKEEVPPASSSD---SEQEDSEENGGDGLKN 198  TZ_T-D7 EDSTVSTDVSPTIPTPVSEEIITPTLQAQTKEEVPPASSSD---SEQEDSEENKDDILKN 198  TZ_T-E8 EDSTLSTDVSPTIPTPVSEEIITPTLQSQTKEEVPPASSSD---SEQEDSEENGGDGLKN 198  TZ_T-C3 EDSTVSTDVSPTIPTPVSEEIITPTLQAQTKEEVPPASSSD---SEQEDSEENGDNVLKN 198  KZN_HIP_D3 EDSTLSTDVSPTIPTPVSEEIITSTLQAQTKEEVPPASSSD---SEQEDSEENGNDGLKN 198  KZN_HIP_B8 EDSTLSTDVSPTIPTPVSEEIITSTLQAQTKEEVPPASSSD---SEQEDSEENGNDGLKN 198  KZN_HIP_D10 EDSTLSTDVSPTIPTPVSEEIITSTLQAQTKEEVPPASSSD---SEQEDSEENGNDGLKN 198  KZN_HIP_E3 EDSTLSTDVSPTIPTPVSEEIITSTLQAQTKEEVPPASSSD---SEQEDSEENGNDGLKN 198  Moz_Buf_5b EDSTVSKDVSPTIPTPVSEEIITPTLQAQTKEEVPPASSSD---SEQEDSEENKDDILKN 198  Moz_Buf_8b EDSTVSKDVSPTIPTPVSEEIITPTLQAQTKEEVPPASSSD---SEQEDSEENKDDILKN 198  Moz_Buf_7 EDSTLSTDVSPTIPTPVSEEIITPTLQTQTKEEIPPKSDSE---SEQEDSEENEDDVLKN 198  Moz_Buf_9 EDSTLSTDVSPTIPTPVSEEIITPTLQTQTKEEIPPKSDSE---SEQEDSEENEDDVLKN 198  Moz_Buf_1c EDSTLSTDVSPTIPTPVSEEIITPTLQTQTEEEIPPKSDSE---SEQEDSEENEDDVLKN 198  Moz_Buf_6c EDSTLSTDVSPTIPTPVSEEIITPTLQTQTEEEIPPKSDSE---SEQEDSEENEDDVLKN 198  **JX442247** EDSTLSTDVSPTIPTPVSEEIIKPTLHTQTKEEIPPKSDSE---SEQEDSEENEDDVLKN 198 | |
| Cattle | **KY912979** GRTDGKNGDAGAKGVGTDGSSSSNGTHSPKKTETSISQ-----PSPGTTTIS-------- 258  **KY912995** GRTDGKNGDAGARGVGTDGSSSSNGIHSPKKTETSISQ-----PSPGTTTIS-------- 258  KNP_MN_C81 GRTDGKKGAAGARGVGTDEFSSSNGAHSPKKSESSINQ-----PSPGTTTLS-------- 258  KNP_MN_C108_4 GRTDGKKGAAGARGVGTDEFSSSNGAHSPKKSESSINQ-----PSPGTTTLS-------- 258  KNP_MN_C89_1 GRTDGKNGAAGARGVGTDGSSSSNGIHSPKKTETSISQ-----PSPGTTTIS-------- 258  KNP_MN_C91 GRTDGKNGAAGARGVGTDGSSSSNGIHSPKKTETSISQ-----PSPGTTTIS-------- 258 | |
| Buffalo | **KY912979** GRTDGKNGDAGAKGVGTDGSSSSNGTHSPKKTETSISQ-----PSPGTTTIS-------- 258  **KY912995** GRTDGKNGDAGARGVGTDGSSSSNGIHSPKKTETSISQ-----PSPGTTTIS-------- 258  **JX442249** GRTDGKNGAAGARGVGTDGSSSSNGIHSPKKTETSISQ-----PSPGTTTIS-------- 258  K_Mar_B6 GRTDGKNGDAGAKGVGTDGSSSSNGTHSPKKTETSISQ-----PSPGTTTIS-------- 258  K_Mar_D2 GRTDGKNGDAGAKGVGTDGSSSSNGTHSPKKTETSISQ-----PSPGTTTIS-------- 258  K_Mar_A4 GRTDGKNGDAGAKGVGTDGSSSSNGTHSPKKTETSISQ-----PSPGTTTIS-------- 258  K_Mar_C2 GRTDGKNGDAGAKGVGTDGSSSSNGTHSPKKTETSISQ-----PSPGTTTIS-------- 258  TZ_T-A10 GRTDGKNGDAGARGVGTDGSSSSNGIHSPKKTETSISQ-----PSPGTTTIS-------- 258  TZ_T-D7 GRTDGKNGDAGAKGVGTDGSSSSNGTHSPKKTETSISQ-----PSPGTTTIS-------- 258  TZ_T-E8 GRTDGKNGDAGARGVGTDGSSSSNGIHSPKKTETSISQ-----PSPGTTTIS-------- 258  TZ_T-C3 GRTDGKNGDAGAKGVGTDGSSSSNGTHSPKKTETSISQ-----PSPGTTTIS-------- 258  KZN_HIP_D3 GRTDGKNGAAGARGVGTDGSSSSNGIHSPKKTETSISQ-----PSPGTTTIS-------- 258  KZN_HIP_B8 GRTDGKNGAAGARGVGTDGSSSSNGIHSPKKTETSISQ-----PSPGTTTIS-------- 258  KZN_HIP_D10 GRTDGKNGAAGARGVGTDGSSSSNGIHSPKKTETSISQ-----PSPGTTTIS-------- 258  KZN_HIP_E3 GRTDGKNGAAGARGVGTDGSSSSNGIHSPKKTETSISQ-----PSPGTTTIS-------- 258  Moz_Buf_5b GRTDGKNGDAGAKGVGTDGSSSSNGTHSPKKTETSISQ-----PSPGTTTIS-------- 258  Moz_Buf_8b GRTDGKNGDAGAKGVGTDGSSSSNGTHSPKKTETSISQ-----PSPGTTTIS-------- 258  Moz_Buf_7 GRTDRKNGAAGDRGVGTDGSSSSNGIHSPKKTETSISQ-----PSPGTTTIS-------- 258  Moz_Buf_9 GRTDRKNGAAGDRGVGTDGSSSSNGIHSPKKTETSISQ-----PSPGTTTIS-------- 258  Moz_Buf_1c GRTDRKNGAAGDRGVGTDGSSSSNGIHSPKKTETSISQ-----PSPGTTTIS-------- 258  Moz_Buf_6c GRTDRKNGAAGDRGVGTDGSSSSNGIHSPKKTETSISQ-----PSPGTTTIS-------- 258  **JX442247** GRTDRKNGTAGARGVGTDGSSSSNGIHSPKKTETSISQ-----PSPGTTTIS-------- 258 | |

**^a^** Reference sequences are bolded. Annotation of other sequence IDs is provided in Figure 1 legend.

**^b^** Amino acid substitutions are highlighted in cyan; TpM12 (TKEEVPPADLSDQVP**)** is a B-cell epitope. AR22.7 (LQPGKTS**)** epitope not found.
