# Supplementary material for: Analysis of p67 allelic sequences reveals a subtype of allele type 1 unique to buffalo-derived Theileria parva parasites from southern Africa
Source: PLoS One. 2020 Jun 29;15(6):e0231434. doi: 10.1371/journal.pone.0231434 (PMC7323972; doi:10.1371/journal.pone.0231434)
Supplement: S4 Table — (DOCX) [file pone.0231434.s005.docx]

**S4 Table.** Predicted protein sequence alignment of allele type 4 identified in *T. parva* parasites from cattle and buffalo

| Host | ^a^Sequence ID/Accession number | ^b^Predicted protein sequence |
| --- | --- | --- |
| Cattle | **TpM12**  KNP_MN_C71 KIAPLSTDVSPTIPTPVSEEIITPTLQAQTKEEVPPASSSD---SEQEDSEENGNDGLKN 198  KNP_MN_F369 EDSSLGTDVPHSIPTPVSEEIITPTLQAQTNEEVPPADLSD---SEQEDSEENEDDTLKN 198  KNP_MN_F369_2 EDSSLGTDVPHSIPTPVSEEIITPTLQAQTNEEVPPADLSD---SEQEDSEENEDDTLKN 198  KNP_MN_F369_1 EDSSLGTDVPHSIPTPVSEEIITPTLQAQTNEEVPPADLSD---SEQEDSEENEDDTLKN 198  KNP_MN_F369_4 EDSSLGTDVPHSIPTPVSEEIITPTLQAQTNEEVPPADLSD---SEQEDSEENEDDTLKN 198 | |
| Buffalo | **JX442251** EDSTLSTDVSPTIPTPVSEEIITPTLQAQTKEEVPPASSSD---SEQEDSEENGNDGLKN 198  TZ_T-D9 EDSTLTTDVSPTIPTPVSEEIITPTLQAQTKEEVPPASSSD---SEQEDSEENGDNVLKN 198  TZ_T-B1 EDSSLGTDVPQSIPTPVSEEIITPTLQAQTKEEVPPASGSD---SEQEDSEENEDDTLKN 198  TZ_T-B9 EDSSLGTDVPQSIPTPVSEEIITPTLQAQTKEEVPPASGSD---SEQEDSEENEDDTLKN 198  TZ_T-A1 EDSSLGTDVPQSIPTPVSEEIITPTLQAQTKEEVPPASGSD---SEQEDSEENEDDTLKN 198  Moz_Buf_10c EDSSLGTDVPHSIPTPVSEEIITPTLQAQTNEEVPPADLSD---SEQEDSEENEDDTLKN 198  **JX442250** EDSSLGTDVPHSIPTPVSEEIITPTLQAQTNEEVPPADLSD---SEQEDSEENEDDTLKN 198 | |
| Cattle | KNP_MN_C71 GRTDGKNGDAGARGVGTDGSSSSNGTHSPKKTETSSNE-----HSPGTTTLSSGV--STS 258  KNP_MN_F369 GRTDGKNGDAGARGVGTDGSSSSNGTHSPKKTETSSNE-----HSPGTTTLSSGV--STS 258  KNP_MN_F369_2 GRTDGKNGDAGARGVGTDGSSSSNGTHSPKKTETSSNE-----HSPGTTTLSSGV--STS 258  KNP_MN_F369_1 GRTDGKNGDAGARGVGTDGSSSSNGTHSPKKTETSSNE-----HSPGTTTLSSGV--STS 258  KNP_MN_F369_4 GRTDGKNGDAGARGVGTDGSSSSNGTHSPKKTETSSNE-----HSPGTTTLSSGV--STS 258 | |
| Buffalo | **JX442251** GRTDGKNGDAGARGVGTDGSSSSNGTHSPTKTETSSNE-----HSPGTTTLSSGV--STS 258  TZ_T-D9 GRTDGKNGDAGAKGVGTDGSSSSNGTHSPKKTETSSNE-----HSPGTTTLSSGV--STS 258  TZ_T-B1 GRTDGKNGDAGARRVGTDGSSSSNGTHSPKKTETSSNE-----HSPGTTTLSSGV--STS 258  TZ_T-B9 GRTDGKNGDAGARRVGTDGSSSSNGTHSPKKTETSSNE-----HSPGTTTLSSGV--STS 258  TZ_T-A1 GRTDGKNGDAGARRVGTDGSSSSNGTHSPKKTETSSNE-----HSPGTTTLSSGV--STS 258  Moz_Buf_10c GRTDGKNGDAGARGVGTDGSSSSNGTHSPKKTETSSNE-----HSPGTTTLSSGV--STS 258  **JX442250** GRTDGKNGDAGARGVGTDGSSSSNGTHSPKKTETSSNE-----HSPGTTTLSSGV--STS 258 | |

**^a^** Reference sequences are bolded. Annotation of other sequence IDs is provided in Figure 1 legend.

**^b^** Amino acid substitutions are highlighted in cyan; TpM12 (TKEEVPPADLSDQVP**)** is a B-cell epitope. AR22.7 (LQPGKTS**)** epitope not found.
