# Supplementary material for: Analysis of p67 allelic sequences reveals a subtype of allele type 1 unique to buffalo-derived Theileria parva parasites from southern Africa
Source: PLoS One. 2020 Jun 29;15(6):e0231434. doi: 10.1371/journal.pone.0231434 (PMC7323972; doi:10.1371/journal.pone.0231434)
Supplement: S5 Table — (DOCX) [file pone.0231434.s006.docx]

**S5 Table.** Estimates of the evolutionary divergence between sequences of allele type 1 from *T. parva* parasites from East and Southern Africa.

| M67476 |  |  |  |  |  |  |  |  |  |  |  |  |  |  |  |  |  |
| --- | --- | --- | --- | --- | --- | --- | --- | --- | --- | --- | --- | --- | --- | --- | --- | --- | --- |
| UG_NK_3g | 0.000 |  |  |  |  |  |  |  |  |  |  |  |  |  |  |  |  |
| UG_MBara_ME6 | 0.000 | 0.000 |  |  |  |  |  |  |  |  |  |  |  |  |  |  |  |
| TZ_TTb5 | 0.000 | 0.000 | 0.000 |  |  |  |  |  |  |  |  |  |  |  |  |  |  |
| TZ_TSb4 | 0.000 | 0.000 | 0.000 | 0.000 |  |  |  |  |  |  |  |  |  |  |  |  |  |
| KE_NKR_8 | 0.000 | 0.000 | 0.000 | 0.000 | 0.000 |  |  |  |  |  |  |  |  |  |  |  |  |
| LK054513 | 0.000 | 0.000 | 0.000 | 0.000 | 0.000 | 0.000 |  |  |  |  |  |  |  |  |  |  |  |
| Zambia_L1 | 0.000 | 0.000 | 0.000 | 0.000 | 0.000 | 0.000 | 0.000 |  |  |  |  |  |  |  |  |  |  |
| KY912962 | 0.000 | 0.000 | 0.000 | 0.000 | 0.000 | 0.000 | 0.000 | 0.000 |  |  |  |  |  |  |  |  |  |
| KY912963 | 0.000 | 0.000 | 0.000 | 0.000 | 0.000 | 0.000 | 0.000 | 0.000 | 0.000 |  |  |  |  |  |  |  |  |
| Moz_Buf_5c | 0.031 | 0.031 | 0.031 | 0.031 | 0.031 | 0.031 | 0.031 | 0.031 | 0.031 | 0.031 |  |  |  |  |  |  |  |
| Moz_Buf_3 | 0.031 | 0.031 | 0.031 | 0.031 | 0.031 | 0.031 | 0.031 | 0.031 | 0.031 | 0.031 | 0.000 |  |  |  |  |  |  |
| KNP_MN_C89_2 | 0.031 | 0.031 | 0.031 | 0.031 | 0.031 | 0.031 | 0.031 | 0.031 | 0.031 | 0.031 | 0.000 | 0.000 |  |  |  |  |  |
| KNP_MN_C108_6 | 0.031 | 0.031 | 0.031 | 0.031 | 0.031 | 0.031 | 0.031 | 0.031 | 0.031 | 0.031 | 0.000 | 0.000 | 0.000 |  |  |  |  |
| KNPW8_44 | 0.031 | 0.031 | 0.031 | 0.031 | 0.031 | 0.031 | 0.031 | 0.031 | 0.031 | 0.031 | 0.000 | 0.000 | 0.000 | 0.000 |  |  |  |
| KNP_MN_C81_3 | 0.031 | 0.031 | 0.031 | 0.031 | 0.031 | 0.031 | 0.031 | 0.031 | 0.031 | 0.031 | 0.000 | 0.000 | 0.000 | 0.000 | 0.000 |  |  |
| KZN_HIP_B9 | 0.090 | 0.090 | 0.090 | 0.090 | 0.090 | 0.090 | 0.090 | 0.090 | 0.090 | 0.090 | 0.104 | 0.104 | 0.104 | 0.104 | 0.104 | 0.104 |  |
| KZN_HIP_B3 | 0.090 | 0.090 | 0.090 | 0.090 | 0.090 | 0.090 | 0.090 | 0.090 | 0.090 | 0.090 | 0.104 | 0.104 | 0.104 | 0.104 | 0.104 | 0.104 | 0.000 |

The number of base substitutions per site from between sequences are shown. Analyses were conducted using the Maximum Composite Likelihood model. The analysis involved 18 nucleotide sequences. Codon positions included were 1^st^+2^nd^+3^rd^+Noncoding. All positions containing gaps and missing data were eliminated. There were a total of 497 positions in the final dataset. Evolutionary analyses were conducted in MEGA7.
