# Supplementary material for: Analysis of p67 allelic sequences reveals a subtype of allele type 1 unique to buffalo-derived Theileria parva parasites from southern Africa
Source: PLoS One. 2020 Jun 29;15(6):e0231434. doi: 10.1371/journal.pone.0231434 (PMC7323972; doi:10.1371/journal.pone.0231434)
Supplement: S6 Table — (DOCX) [file pone.0231434.s007.docx]

**S6 Table.** Taxonomic metadata detailing the grouping of p67 allele types from *T. parva* parasites from East and Southern Africa.

| ^a^Sequence ID | ^b^Accession number | Country of origin | Locality | Host | Year collected | Associated disease | Reference | Tree group |
| --- | --- | --- | --- | --- | --- | --- | --- | --- |
| UG_NK_3g | MT199341 | Uganda | Karamoja | Cattle | 2017 | ECF | Current study | Allele type 1  Group 1 |
| UG_Mbara_ME6 | MT199342 | Uganda | Mbarara | Cattle | 2017 | ECF | Current study |  |
| TZ_TTb5 | MT199343 | Tanzania | Tanga | Cattle | 2014 | ECF | Current study |  |
| TZ_TSb4 | MT199344 | Tanzania | Simanjiro | Cattle | 2014 | ECF | Current study |  |
| M67476 | M67476 | Kenya | Kilifi | Cattle | 1992 | ECF | Nene *et al*., 1992 |  |
| KE_NKR_8 |  | Kenya | Nakuru | Cattle | 2017 | ECF | Current study |  |
| Marula_10 | LK054513 | Kenya | Nakuru | Cattle | 2014 | CD | Obara *et al*., 2015 |  |
| ILRI-1 | KY912962 | Kenya | Laikipia | Cattle | 2015 | CD | Sitt *et al*., 2019 |  |
| ILRI-2 | KY912963 | Kenya | Laikipia | Buffalo | 2015 | CD | Sitt *et al*., 2019 |  |
| Zambia_L1 |  | Zambia | Zambia | Cattle | 2009 | ECF | Sibeko *et al*., 2010 |  |
| Moz_Buf_5c | MT199345 | Mozambique | Marromeu Game Reserve | Buffalo | 2016 | CD | Current study | Allele type 1  Group 2 |
| Moz_Buf_3 |  | Mozambique | Marromeu Game Reserve | Buffalo | 2016 | CD | Current study |  |
| KNP_MN_C81_3 |  | South Africa | Bushbackridge (Mnisi) | Cattle | 2013 | CD | Current study |  |
| KNP_MN_C89_2 | MT199346 | South Africa | Bushbackridge (Mnisi) | Cattle | 2013 | CD | Current study |  |
| KNP_MN_C108_6 |  | South Africa | Bushbackridge (Mnisi) | Cattle | 2013 | CD | Current study |  |
| KNPW8_44 |  | South Africa | Kruger National Park | Buffalo | 2009 | CD | Sibeko KP, unpublished |  |
| KNPW8_17 |  | South Africa | Kruger National Park | Buffalo | 2009 | CD | Sibeko KP, unpublished |  |
| Mab BB43_2 |  | South Africa | Limpopo | Buffalo | 2009 | CD | Sibeko KP, unpublished |  |
| KZN_HIP_B3 | MT199347 | South Africa | Hluhluwe-iMfolozi Park | Buffalo | 2017 | CD | Current study | Allele type 1  Group 3 |
| KZN_HIP_B9 |  | South Africa | Hluhluwe-iMfolozi Park | Buffalo | 2017 | CD | Current study |  |
| KNP2 | AF079177 | South Africa | Kruger National Park | Buffalo | 1999 | CD | Nene *et al*., 1999 |  |
| TZ_TE1 | MT199350 | Tanzania | Tanga | Buffalo | 2014 | CD | Current study | Allele type 2  Group 1 |
| 7014 | U40703 | Kenya | Kilifi | Buffalo | 1996 | CD | Nene *et al.,*1996 |  |
| K_Mar_D8 |  | Kenya | Nakuru | Cattle | 2014 | CD | Current study |  |
| ILRI-4 | KY912965 | Kenya | Laikipia | Cattle, Buffalo | 2015 | CD | Sitt *et al*., 2019 |  |
| ILRI-6 | KY912967 | Kenya | Laikipia | Cattle, Buffalo | 2015 | CD | Sitt *et al*., 2019 |  |
| Marula_2 | LK054505 | Kenya | Nakuru | Cattle | 2014 | CD | Obara *et al*., 2015 |  |
| Moz_Buf_10 | MT199348 | Mozambique | Marromeu Game Reserve | Buffalo | 2016 | CD | Current study | Allele type 2  Group 2 |
| KZN_HIP_E6 | MT199349 | South Africa | Hluhluwe-iMfolozi Park | Buffalo | 2017 | CD | Current study |  |
| KZN_HIP_D7 |  | South Africa | Hluhluwe-iMfolozi Park | Buffalo | 2017 | CD | Current study |  |
| KNP_MN_C108_9 |  | South Africa | Bushbackridge (Mnisi) | Cattle | 2013 | CD | Current study |  |
| K_Mar_A7 |  | Kenya | Nakuru | Buffalo | 2014 | CD | Current study |  |
| K_Mar_E1 |  | Kenya | Nakuru | Buffalo | 2014 | CD | Current study |  |
| TZ_TA1 | MT199356 | Tanzania | Tanga | Buffalo | 2014 | CD | Current study | Allele type 4 |
| Moz_Buf_10c | MT199355 | Mozambique | Marromeu Game Reserve | Buffalo | 2016 | CD | Current study |  |
| KNP_MN_F369 | MT199354 | South Africa | Bushbackridge (Mnisi) | Cattle | 2017 | CD | Current study |  |
| KNP102-26 | JX442250 | South Africa | Kruger National Park | Buffalo | 2009 | CD | Sibeko *et al*., 2010 |  |
| TZ_TD9 |  | Tanzania | Tanga | Buffalo | 2014 | CD | Current study |  |
| KNPW8-48 | JX442251 | South Africa | Kruger National Park | Buffalo | 2009 | CD | Sibeko *et al*., 2010 |  |
| KNP_MN_C71 |  | South Africa | Bushbackridge (Mnisi) | Cattle | 2013 | CD | Current study |  |
| TZ_TE8 |  | Tanzania | Tanga | Buffalo | 2014 | CD | Current study | Allele type 3 |
| TZ_TC3 | MT199353 | Tanzania | Tanga | Buffalo | 2014 | CD | Current study |  |
| K_Mar_A4 |  | Kenya | Nakuru | Buffalo | 2014 | CD | Current study |  |
| ILRI-18 | KY912979 | Kenya | Laikipia | Cattle, Buffalo | 2015 | CD | Sitt *et al*., 2019 |  |
| Marula_7 | LK054510 | Kenya | Nakuru | Cattle | 2014 | CD | Obara *et al*., 2015 |  |
| Moz_Buf_5b |  | Mozambique | Marromeu Game Reserve | Buffalo | 2016 | CD | Current study |  |
| KZN_HIP_E3 |  | South Africa | Hluhluwe-iMfolozi Park | Buffalo | 2017 | CD | Current study |  |
| KNP_MN_C89_1 |  | South Africa | Bushbackridge (Mnisi) | Cattle | 2013 | CD | Current study |  |
| KNPW8-35 | JX442249 | South Africa | Kruger National Park | Buffalo | 2009 | CD | Sibeko *et al*., 2010 |  |
| ILRI-34 | KY912995 | Kenya | Laikipia | Cattle, Buffalo | 2015 | CD | Sitt *et al*., 2019 |  |
| Moz_Buf_9 | MT199351 | Mozambique | Marromeu Game Reserve | Buffalo | 2016 | CD | Current study |  |
| KNP102-9 | JX442247 | South Africa | Kruger National Park | Buffalo | 2009 | CD | Sibeko *et al*., 2010 |  |
| KNP_MN_C108_4 |  | South Africa | Bushbackridge (Mnisi) | Cattle | 2013 | CD | Current study |  |
| KNP_MN_C81 | MT199352 | South Africa | Bushbackridge (Mnisi) | Cattle | 2013 | CD | Current study |  |

**^a^** Reference sequence IDs are bolded.

**^b^** Sequences from the current study will be released to the GenBank public database after publication.
